# Plasma Albumin Redox State Is Responsive to the Amino Acid Balance of Dietary Proteins in Rats Fed a Low Protein Diet

**DOI:** 10.3389/fnut.2019.00012

**Published:** 2019-02-15

**Authors:** Yasuaki Wada, Xijier, Namiko Seto, Yosuke Komatsu, Muneya Tsuda, Yohei Kitamura, Hirohisa Izumi, Takashi Shimizu, Yasuhiro Takeda

**Affiliations:** Wellness & Nutrition Science Institute, Morinaga Milk Industry Co. Ltd., Zama, Japan

**Keywords:** amino acid balance, glutathione, low protein diet, mercaptalbumin, non-mercaptalbumin, plasma albumin, quality of dietary protein, redox state of plasma albumin

## Abstract

We recently reported that plasma albumin redox state, which correlates with albumin synthesis rate, could be associated with the quality of dietary protein. Aiming to elucidate the association between them, plasma albumin redox state was investigated in rats fed various kinds of AIN-93G-based low protein diets. Plasma albumin redox state was shifted to a more oxidized state in rats fed 3% casein (CN) diet than those fed 3% whey protein or 3% wheat gluten diet, while supplementing 3% CN diet with cystine reversed it to a more reduced state, indicating that cystine would complement the shortage of cysteine in CN, thereby increasing albumin synthesis rate. Supplementation with glutathione, a cysteine-containing antioxidative tripeptide, normalized hepatic glutathione redox state modulated by ingestion of 3% CN diet, but it only reversed the oxidized shift of plasma albumin redox state to an extent similar to cystine alone or the constituting amino acid mixture of glutathione (i.e., glutamic acid, cystine, and glycine), indicating that glutathione would primarily serve as a source of cysteine rather than exert its antioxidative activity. Plasma albumin would thus be influenced by amino acid balance in dietary proteins, and it could be useful as a biomarker that contributes to prevention of protein under-nutriton, caused by not only insufficient protein intake but also ingestion of poor-quality protein.

## Introduction

Albumin comprises the largest part of proteins in plasma of mammals (1). This protein has three isoforms according to the redox states of a free cysteine (Cys) residue at position 34 (2). Albumin with a free thiol on Cys34 is the reduced form and designated as mercaptalbumin (MA), while the isoforms with oxidized Cys34, such as a mixed disulfide with low molecular weight thiols and sulfinic/sulfonic acid, are the oxidized forms, which are designated as non-mercaptalbumin (NA)-1 and NA-2, respectively. More than 70% of total plasma albumin is MA in healthy adults (3), but the ratio of MA to total albumin falls in response to physiological and pathophysiological conditions. Many studies have shown the relationship between albumin redox state and liver failure (4, 5). The MA ratio decreased in chronic liver patients, which is likely explained by impaired albumin synthesis and oxidative stress, caused by hepatic disorder. The decrease of MA ratio was alleviated by oral branched-chain amino acid supplementation, possibly because the supplementation would galvanize *de novo* albumin synthesis, thereby complementing the fall of MA. Similar to liver diseases, decreased MA ratio has been reported in patients with renal failure, another oxidative stress-related disease (6, 7). The decrease of MA ratio correlated with impairment in renal function, and the decrease was ameliorated after hemodialysis that may restore antioxidative potential in renal patients. Furthermore, decreased MA ratio has been observed in aging (8), diabetes (9), and strenuous exercise (10–12), all of which may also be attributed to oxidative stress.

We have consecutively reported that the redox state of plasma albumin (the balance of reduced and oxidized forms of plasma albumin) is influenced by the amount of protein intake in rats (13–15), and the ratio of MA to total albumin correlates with the albumin fractional synthesis rate, which is also stimulated by dietary proteins (14). As the redox state of plasma albumin was more responsive to moderate protein insufficiency compared with plasma albumin level, a conventional marker of protein nutritional status (2), it would serve as a robust marker demonstrating potential protein undernutrition (15). Furthermore, we have recently shown that the shift of plasma albumin redox state to an oxidized state in growing rats, induced by ingestion of a casein (CN)-based low protein (LP) diet, was ameliorated when cystine (Cyss) was added to the CN-based LP diet (16). As sulfur amino acids are limiting amino acids for growth and maintenance of rats when they are placed on CN as a protein source (17), it was considered that the redox state of plasma albumin would be shifted to a more oxidized state by dietary amino acid imbalance such as shortage of Cys in CN. Thus, it was suggested that plasma albumin redox state could also be associated with the quality of dietary protein such as amino acid balance. However, the above recent study of ours was conducted only in a short period of time (2 weeks), and energy and protein intakes differed significantly between some of the dietary groups (16). A longer-term study with the feeding of equal amount of protein sources is likely necessary to substantiate the association between protein quality and plasma albumin redox state.

The aim of this study was to elucidate the role of protein quality in determining the redox state of plasma albumin, by focusing on amino acid balance in diet. Growing rats were subjected to three kinds of dietary experiments based on our previous study (14), where animals fed low protein diet initially manifested a decrease in plasma MA ratio and then exhibited hypoalbuminemia. It was therefore considered that this animal model clearly expresses the progression of protein undernutrition could be useful to indicate the difference in the quality of dietary proteins. In Experiment 1, effects of protein sources with different amino acid compositions on the redox state of plasma albumin was investigated under a pair-feeding regimen, such as CN, whey protein (WP), or wheat gluten (WG). CN and WP are both of milk origin. They differ significantly in amino acid balance and gastrointestinal digestive motility (18, 19), while both have been reported to have excellent protein digestibility as assessed by ileal digestibility in pigs and rats (20–22). In contrast, WG is quite inferior to CN in protein quality as determined by the indicator amino acid oxidation (IAAO) method in rats (23, 24). In Experiment 2, the role of limiting amino acids in determining plasma albumin redox state was scrutinized, by placing animals initially on a CN-based low protein diet without Cyss supplementation, and subsequently maintaining them on the diet supplemented with Cyss; we examined whether Cyss supplementation during the last 2 weeks would improve dietary amino acid balance, thereby alleviating oxidized shift of plasma albumin. In Experiment 3, effects of dietary Cys were further explored by supplementing it in the form other than Cyss. Among the dietary Cys supplements, glutathione (γ-L-glutamy-L-cysteinyl-glycine, GSH) was selected, as it is the most abundant intracellular non-protein thiol tripeptide and defends against oxidative stress (25); it was hypothesized that dietary GSH and its disulfide form (GSSG) could modulate the redox state of plasma albumin by exerting its antioxidative activity in addition to serving as a source of Cys to complement its shortage in CN.

## Materials and Methods

### Animal Experiments

This study design was approved by the Animal Research Committee of Morinaga Milk Industry Co., Ltd., and the study was performed in accordance with the committee's guideline. Male Wistar rats (3 weeks old; Japan SLC, Hamamatsu, Japan) were individually placed in polycarbonate cages with wood shavings in a light-, temperature-, and humidity-controlled facility (21–25°C, 40–60% humidity, and 12–h light/dark cycle), and allowed *ad libitum* access to water and AIN-93G diet containing 20% CN (Oriental Yeast, Tokyo, Japan; Table 1). After 1 week of acclimation, animals were subjected to 3 kinds of experiments.

**Table 1:** Composition of experimental diets in Experiments.

	**CT (AIN-93G)**	**% CN^c^**	**3% CN + Cyss**	**3% WP**	**3% WG**
**[INGREDIENT]**	**(g/kg diet)**				
Casein	200	30	30	0	0
Whey protein isolate	0	0	0	28	00
Wheat gluten	0	0	0	0	31
L-Cystine	3	0	0.45	0	0
Cornstarch	397	421	421	423	415
Dextrinized cornstarch	132	281	281	281	281
Sucrose	100	100	100	100	100
Cellulose	50	50	50	50	50
Soybean oil	70	70	70	70	70
*t*-Butylhydroquinone	0.014	0.014	0.014	0.014	0.014
AIN-93 mineral mixture	35	0	0	0	35
Modified mineral mmixture^a^	0	35	35	35	0
AIN-93 vitamin mixture	10	10	10	10	10
Choline BITARTRATE	2.5	2.5	2.5	2.5	2.5
**[NUTRIENTS]**	**(g/kg diet)**				
Energy^b^	15397.1	15418.0	15418.0	15422.2	15455.7
Protein	180.7	28.3	27.9	27.4	27.2
Isoleucine	9.20	1.40	1.40	1.74	1.15
Leucine	16.0	2.40	2.40	3.95	2.18
Lysine	13.69	2.08	2.08	3.20	0.49
Methione	5.10	0.80	0.80	0.70	0.46
Cyss	3.80	0.10	0.60	1.02	0.64
Phenylalanine	8.70	1.30	1.30	1.06	1.57
Tyrosine	9.50	1.40	1.40	1.13	1.05
Threonine	7.10	1.10	1.10	1.44	0.76
Tryptophan	2.10	0.30	0.30	0.71	0.26
Valine	11.50	1.70	1.70	1.53	1.15
Histidine	5.10	0.80	0.80	0.56	0.64
Arginine	6.30	0.90	0.90	0.70	1.03
Alanine	5.10	0.80	0.80	1.54	0.76
Asparatic Acid	12.00	1.80	1.80	3.50	0.95
Glutamic Acid	36.20	5.40	5.40	5.03	10.08
Glycine	3.10	0.50	0.50	0.48	1.01
Proline	19.70	3.00	3.00	1.22	3.44
Serine	8.90	1.30	1.30	1.07	1.40

a *AIN-93G mineral mixture was modified, replacing part of the CaCO_3_ with CaHPO_4_, to make Ca/P ratios in the diets close to that of AIN-93G*.

b *kJ/kg diet*.

c *3% CN diet was supplemented with glutathione (1.15 g/kg), glutathione disulfide (1.15 g/kg), or glutamic acid (0.56 g/kg) + cystine (0.45 g/kg) + glycine (0.29 g/kg), to formulate 3% CN + GSH, 3% CN + GSSG, or 3% CN + Glu/Cyss/Gly diet*.

*CN, CT, Cyss, Glu, Gly GSH, and GSSG, WG, and WP denote casein, control, cystine, glutamic acid, glycine, glutathione, glutathione disulfide, wheat gluten, and whey protein, respectively*.

#### Experiment 1

Thirty animals were assigned to 5 dietary groups (*n* = 6); 1 group was fed AIN-93G as a control diet (20% CN, Oriental Yeast, Tokyo, Japan) (17), and the other 4 were fed an AIN-93G-based iso-energetic LP diet, containing either 3% CN, 3% CN supplemented with Cyss, 3% WP (Morinaga Milk Industry, Tokyo, Japan), or 3% WG (Wako Pure Chemical, Osaka, Japan). These diets were designated as the control (CT) diet, 3% CN diet, 3% CN + Cyss diet, 3% WP diet, and 3% WG diets, respectively, and their compositions are shown in Table 1. While the CT and 3% CN diet groups were fed each diet *ad libitum*, the other groups were pair-fed with the 3% CN diet group. These 5 dietary groups were maintained for 4 weeks. Approximately 50–200 μL of blood samples were drawn from the lateral tail vein once a week, using syringes treated with ethylenediaminetetraacetic acid disodium. Blood samples were centrifuged at 1,700 g for 10 min at room temperature (RT), and the upper plasma layers were obtained and stored at −80°C until analysis.

After 4 weeks of the above dietary regimen, animals were euthanized by deep anesthesia with sevoflurane (Mylan, Canonsburg, PA). Blood was drawn from the inferior vena cava, and plasma layers were obtained after centrifugation. Liver samples were also excised and frozen immediately in liquid nitrogen. These samples were stored at −80°C until analysis.

#### Experiment 2

Eighteen animals were assigned to 3 dietary groups (*n* = 6), and were maintained for 6 weeks as follows. During the first 4 weeks, these groups were initially fed the CT diet, 3% CN diet, or 3% CN + Cyss diet, (Table 1); the CT and 3% CN diet groups were fed *ad libitum*, while the 3% CN + Cyss diet group was pair-fed with the 3% CN diet group. In the subsequent 2 weeks, the CT diet group was fed the control diet *ad libitum* as before, and both groups initially placed on the 3% CN diet and 3% CN + Cyss diet were allowed *ad libitum* access to the 3% CN + Cyss diet. For clarity, these groups were designated as the 3% CN wo/w Cyss diet group and the 3% CN w/w Cyss diet group, respectively. Similar to Experiment 1, blood samples were drawn once a week, and plasma samples were obtained after centrifugation and stored at −80°C until analysis.

After 6 weeks of the above dietary treatments, similar to Experiment 1, animals were euthanized, and plasma and liver samples were obtained and stored at −80°C.

#### Experiment 3

Eighteen animals were assigned to 6 dietary groups (*n* = 3); they were fed the CT diet, 3% CN diet, 3% CN + Cyss diet, or 3% CN diets with 3 kinds of supplements, GSH, GSSG, or an amino acid mixture that constitutes GSH [i.e., glutamic acid (Glu), Cyss, and glycine (Gly)]. These diets were designated as the 3% CN + GSH diet, 3% CN + GSSG diet, and 3% CN + Glu/Cyss/Gly diet, respectively (Table 1). Cyss, GSH, and GSSG supplements contained an equimolar of Cys, and GSH, GSSG, and Glu/Cyss/Gly supplements had equimolars of Glu, Cyss, and Gly. These 6 dietary groups were fed *ad libitum* for 4 weeks, and were then euthanized. Blood was obtained by cardiac puncture, and plasma layers were obtained after centrifugation. Liver samples were also excised and frozen immediately in liquid nitrogen. Both plasma and liver samples were stored at −80°C until analysis.

### Blood Chemistry

Plasma samples were applied to an amino acid analyzer (L-8900; Hitachi High-Technologies, Tokyo, Japan) to analyze free amino acid patterns, as described previously (14, 15). Plasma samples were also subjected to a bromocresol green method (A/G B test kit, Wako Pure Chemical) to determine the levels of albumin.

### Redox State of Plasma Albumin

The redox state of plasma albumin was determined as described previously (14, 15). Albumin isoforms, MA, NA-1, and NA-2 were separated using a Shodex Asahipak ES-502N 7C column (Showa Denko, Kawasaki, Japan). They were eluted using a 100-min gradient with increasing ethanol concentrations from 0 to 10% in 0.4 M sodium sulfate and 50 mM sodium acetate (pH 4.85), with a flow rate of 0.5 mL/min. Fluorescence emission was measured at 280 nm for excitation and 340 nm for emission.

### Hepatic Gene Expression

RNA was extracted from liver samples and real-time PCR was performed with an ABI PRISM 7500 fast real-time PCR system (Applied Biosystems, Foster City, CA) as described previously (14, 15). The reaction was performed using TaqMan universal master mix [No AmpErase UNG (2×)] with the PCR primer and probe set for the genes of albumin and a eukaryotic translation initiation factor 4E-binding protein 1 (4E-BP1) (TaqMan Gene Expression Assays; Rn00592480_m1 and Rn00587824_m1, respectively). Gene expressions were normalized to an endogenous control gene, β-actin (Rn00667869_m1).

### Hepatic GSH/GSSG Level

Total GSH and GSSG levels in liver samples were determined using a GSSG/GSH Quantification Kit (Dojindo Molecular Technologies, Kumamoto, Japan).

### Statistical Analyses

Values are expressed as means ± SD (Experiment 1 and 2, *n* = 6; Experiment 3, *n* = 3). Data were analyzed by one-way ANOVA followed by a Tukey-Kramer HSD test (JMP software, version 5.1.1; SAS Institute, Cary, NC). Significance was demonstrated at *P* < 0.05.

## Results

### Experiment 1

Three kinds of protein sources with different amino acid compositions (CN, WP, and WG) were investigated, by formulating AIN-93G-based LP diets containing each of these protein sources (3% CN, 3% WP, or 3% WG diet). Similar to the case of AIN-93G (20% CN diet), where Cyss is fortified because of the shortage of Cys in CN (17), a 3% CN diet supplemented with Cyss was also formulated (3% CN + Cyss diet), with the CN/Cyss ratio adjusted to that of AIN-93G. Animals were placed on one of these LP diets under a pair-feeding regimen for 4 weeks.

#### Dietary Intake and Body Weight

Energy and protein intakes were significantly higher in the CT diet group than the LP diet groups, and no significant difference was seen between the LP diet groups (Table 2). Body weight of the CT group at 4 weeks was significantly higher than those of the LP diet groups, and the levels did not differ significantly between the LP diet groups (Table 2).

**Table 2:** Energy intake, protein intake, body weight, and plasma free amino acid levels in Experiment 1.

	**CT**	**3% CN**	**3% CN + Cyss**	**3% WP**	**3% WG**
Energy intake (kJ/d)	220.2 ± 23.4^a^	122.1 ± 14.1^b^	120.9 ± 11.4^b^	122.4 ± 15.1^b^	118.0 ± 9.6^b^
Protein intake (g/d)	2.58 ± 0.27^a^	0.22 ± 0.03^b^	0.22 ± 0.02^b^	0.22 ± 0.03^b^	0.21 ± 0.02^b^
**BODY WEIGHT (g)**					
Week 0	84.2 ± 5.2	84.4 ± 4.8	84.7 ± 3.7	84.6 ± 3.8	84.5 ± 3.8
Week 4	228.4 ± 21.5^a^	78.5 ± 6.2^b^	86.8 ± 5.8^b^	87.9 ± 4.9^b^	92.1 ± 5.8^b^
**PLASMA AMINO ACIDS (μmol/mL)**					
Total	4.34 ± 0.70^a^	3.66 ± 0.34^ab^	3.40 ± 0.23^bc^	2.87 ± 0.58^c^	2.94 ± 0.24^bc^
Essential	1.79 ± 0.32^a^	0.99 ± 0.06^b^	0.80 ± 0.07^bc^	0.64 ± 0.12^cd^	0.52 ± 0.08^d^

*Data are expressed as means ± SD (n = 6), and values with different superscript letters are significantly different (P < 0.05). CN, CT, Cyss, WG, and WP denote casein, control, cystine, wheat gluten, and whey protein, respectively*.

#### Blood Chemistry

Patterns of free amino acid were analyzed for plasma samples obtained at the end of the experimental period, as plasma free amino acid levels reflect immediate intakes of dietary proteins and amino acids (26). Both total and essential amino acid levels were lower in all the LP diet groups compared with the CT diet groups, and most of the differences were statistically significant (Table 2). There were also significant differences in these levels between some of the LP diet groups, but they differed to limited extents.

**Figure 1:**
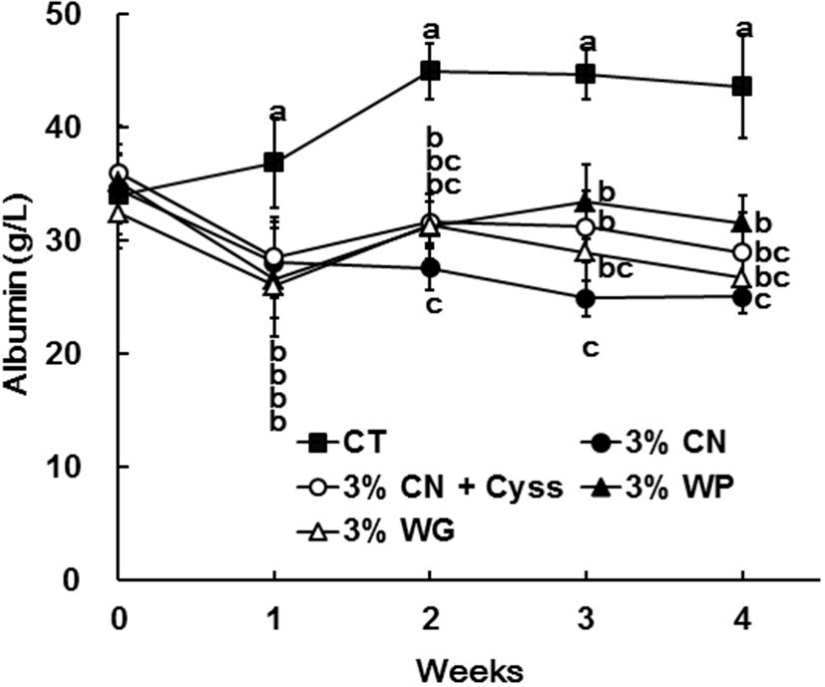
Plasma albumin levels in Experiment 1. The levels of albumin were measured for plasma samples obtained during the experimental period. Data are shown as means ± SD (*n* = 6); values with different letters are significantly different at each time point (*P* < 0.05). CN, CT, Cyss, WG, and WP denote casein, control, cystine, wheat gluten, and whey protein, respectively.

The levels of albumin were determined for plasma samples obtained during the experimental period. Plasma albumin levels in all the LP diet groups initially decreased, and remained constant thereafter (Figure 1). The decreased plasma albumin levels in the LP diet groups were significantly lower than the levels of CT diet group. Plasma albumin level of the 3% CN diet group were the lowest among the LP diet groups, and was significantly lower than the 3% WP diet group.

#### Redox State of Plasma Albumin

**Figure 2:**
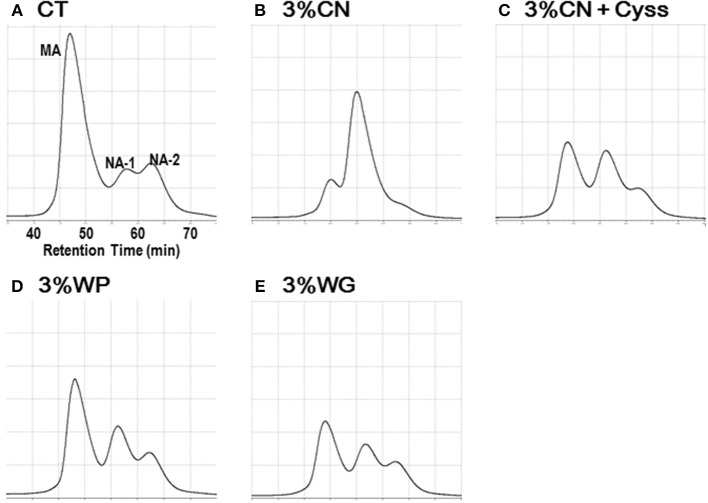
Chromatograms of plasma albumin in Experiment 1. Plasma samples, obtained during the experimental period, were subjected to HPLC to determine albumin redox state. Typical chromatograms of the **(A)** control diet (CT diet), **(B)** 3% casein diet (3% CN diet), **(C)** 3% CN diet supplemented with cystine, (3% CN + Cyss diet), **(D)** 3% whey protein diet (3% WP diet), and **(E)** 3% wheat gluten diet (3% WG diet) groups, at week 4 are shown. MA and NA denote mercaptalbumin and non-mercaptalbumin, respectively.

**Figure 3:**
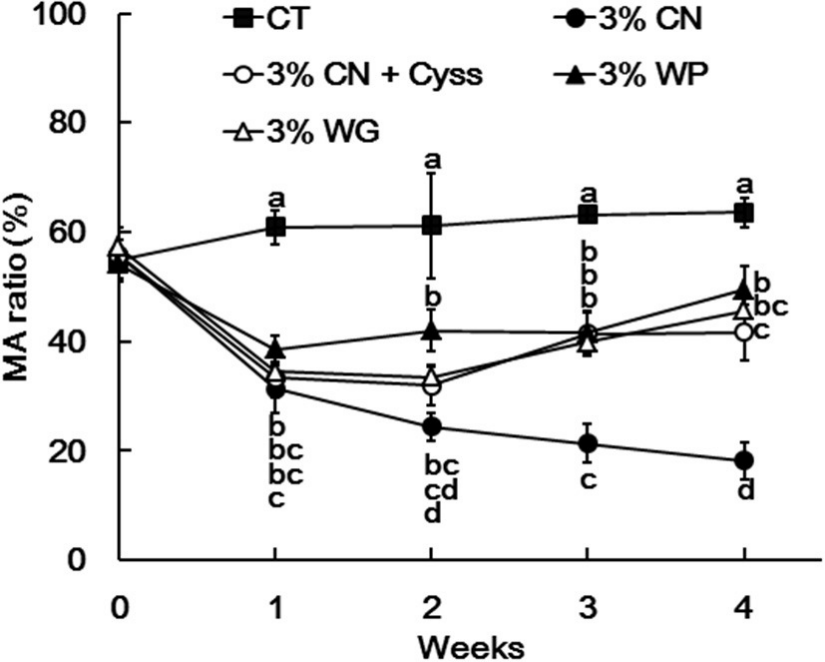
Ratio of mercaptalbumin among total plasma albumin in Experiment 1. Plasma samples, obtained weekly, were subjected to HPLC to determine albumin redox state. Ratios of mercaptalbumin (MA) among total albumin are shown as means ± SD (*n* = 6); values with different letters are significantly different at each time point (*P* < 0.05). CN, CT, Cyss, WG, and WP denote casein, control, cystine, wheat gluten, and whey protein, respectively.

Plasma samples, obtained during the experimental period, were analyzed to determine the albumin redox state (Figure 2). As was seen in our previous studies (13, 14, 16), plasma albumin redox state shifted to a more oxidized states in all the LP diet groups. When it was expressed as the ratio of MA among total albumin, the ratio in the LP diet groups dropped rapidly early in the experimental period (Figure 3). Particularly, MA ratio of the 3% CN diet group was the lowest among the LP diet groups, and the ratio continued to decline until the end of the experimental period.

#### Hepatic Gene Expression

**Figure 4:**
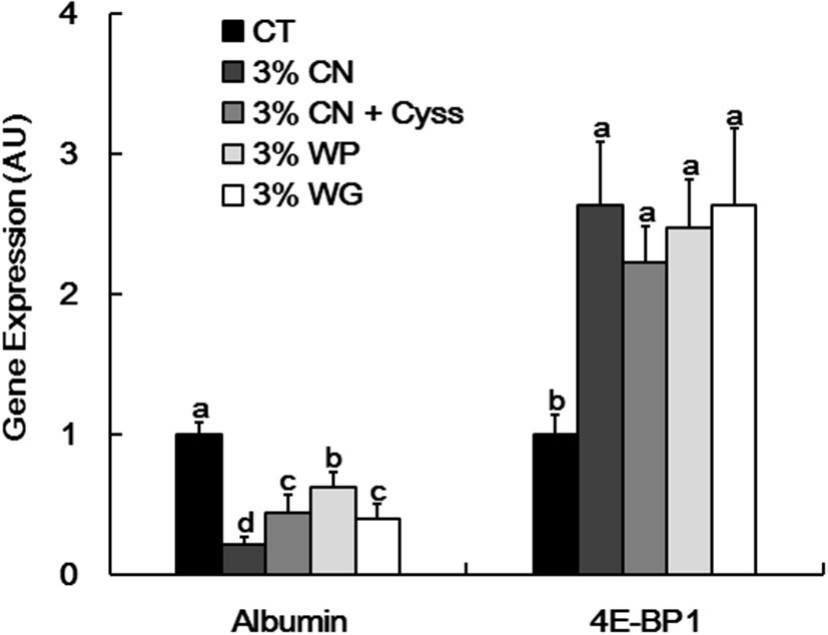
Hepatic albumin and 4E-BP1 gene expression in Experiment 1. Gene expression of albumin and eukaryotic initiation factor 4E-binding protein 1 (4E-BP1) was determined in livers obtained at the end of the experimental period. Data are shown as means ± SD (*n* = 6), and values with different letters are significantly different (*P* < 0.05). CN, CT, Cyss, WG, and WP denote casein, control, cystine, wheat gluten, and whey protein, respectively.

As albumin synthesis is primarily regulated at the transcription level (2), albumin gene expression in livers was examined at the end of the experimental period. Albumin expression was significantly suppressed in the LP diet groups compared with the CT diet group (Figure 4). In particular, the expression level was the lowest in 3% CN diet group among the LP diet groups.

Our recent study showed that 4E-BP1, an eukaryotic translation initiation factor, was induced at both transcriptional and translational levels in the livers of young rats placed on a CN-based LP diet, thereby suppressing downstream protein synthesis including albumin; lower Cys content in CN is likely responsible for the 4E-BP1 induction (16). As MA ratio correlates with albumin synthesis rate (14), it is considered that this Cys-mediated regulatory system could also modulate the MA ratio of the LP diet groups. Compared with the CT diet group. 4E-BP1 gene was significantly induced in all the LP diet groups (Figure 4). However, no significant difference in this gene expression was seen between the LP diet groups.

### Experiment 2

To further confirm how limiting amino acids would specify plasma albumin redox state, focusing on the shortage of Cys in CN, rats were initially placed on the 3% CN diet or 3% CN + Cyss diet under a pair-feeding regimen for 4 weeks, and both groups were then fed 3% CN diet + Cyss *ad libitum* for 2 weeks (these groups were designated as the 3% CN wo/w Cyss diet group and the 3% CN w/w Cyss diet group, respectively). It was investigated how plasma albumin redox state in the 3% CN wo/w Cyss diet group was shifted during the last 2 weeks.

#### Dietary Intake and Body Weight

During the first 4 weeks, similar to Experiment 1, energy and protein intakes were not significantly different between the 3% CN wo/w Cyss diet and 3% CN diet w/w Cyss diet groups, as they were maintained on a pair-feeding regimen (Table 3). In the following 2 weeks, when both of these groups were fed the 3% CN diet + Cyss diet *ad libitum*, the 3% CN wo/w Cyss diet group showed decreased energy intake that was significantly lower than the 3% CN w/w Cyss diet group. Protein intake was also lower in the 3% CN wo/w Cyss diet group at week 6, but the difference did not reach statistical significance.

**Table 3:** Energy intake, protein intake, body weight, and plasma free amino acid levels in Experiment 2.

	**CT**	**3% CN wo/w Cyss**	**3% CN w/w Cyss**
**ENERGY INTAKE (kJ/d)**			
Week 0–4	223.6 ± 7.1^a^	135.7 ± 8.5^b^	136.0 ± 8.5^b^
Week 4–6	250.4 ± 12.2^a^	113.3 ± 8.9^c^	133.6 ± 15.8^b^
**PROTEIN INTAKE (g/d)**			
Week 0–4	2.62 ± 0.08^a^	0.25 ± 0.02^b^	0.25 ± 0.02^b^
Week 4–6	2.94 ± 0.14^a^	0.21 ± 0.02^b^	0.24 ± 0.03^b^
**BODY WEIGHT (g)**			
Week 0	84.6 ± 3.4	84.6 ± 3.4	84.5 ± 3.2
Week 4	222.5 ± 5.8^a^	79.4 ± 3.1^b^	91.9 ± 3.5^c^
Week 6	269.4 ± 6.3^a^	84.6 ± 3.4^b^	101.6 ± 4.7^c^
**PLASMA AMINO ACIDS (μmol/mL)**			
Total	4.48 ± 0.36^a^	3.49 ± 0.31^b^	3.28 ± 0.35^b^
Essential	1.84 ± 0.22^a^	0.79 ± 0.09^b^	0.75 ± 0.10^b^

*Data are expressed as means ± SD (n = 6), and values with different superscript letters are significantly different (P < 0.05). CN, CT, and Cyss denote casein, control, and cystine, respectively*.

There was a marked difference in body weights between the 3% CN wo/w Cyss diet group and the 3% CN w/w Cyss diet group; it was significantly lower in the former than the latter at week 4, and the statistical significance persisted even after they were both fed the 3% + Cyss diet *ad libitum* for 2 weeks (Table 3).

#### Blood Chemistry and Redox State of Plasma Albumin

Free amino acid patterns of plasma samples, obtained at the end of the experimental period (at week 6), were analyzed. Neither total amino acid levels nor essential amino acid levels were significantly different between the two LP diet groups (Table 3).

**Figure 5:**
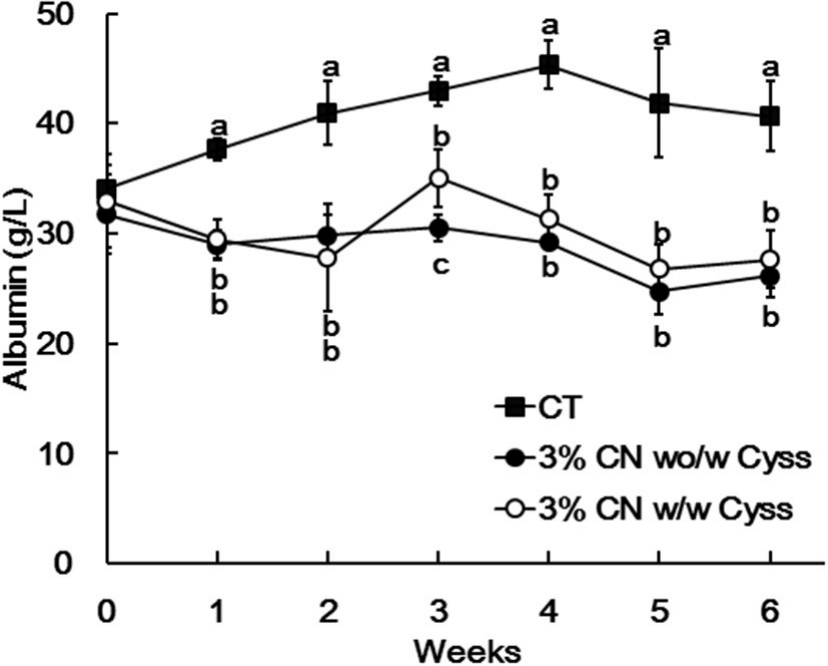
Plasma albumin levels in Experiment 2. The levels of albumin were measured for plasma samples obtained during the experimental period. Data are shown as means ± SD (*n* = 6); values with different letters are significantly different at each time point (*P* < 0.05). CN, CT, and Cyss denote casein, control, and cystine, respectively.

Plasma albumin levels were determined during the experimental period. The levels of the 3% CN wo/w Cyss diet group tended to be lower than those of 3% CN w/w Cyss diet group throughout the experimental period, but it was only significant at week 3 (Figure 5). Furthermore, this switch of dietary regimen had little impact on the plasma albumin level of the 3% CN wo/w Cyss diet group.

**Figure 6:**
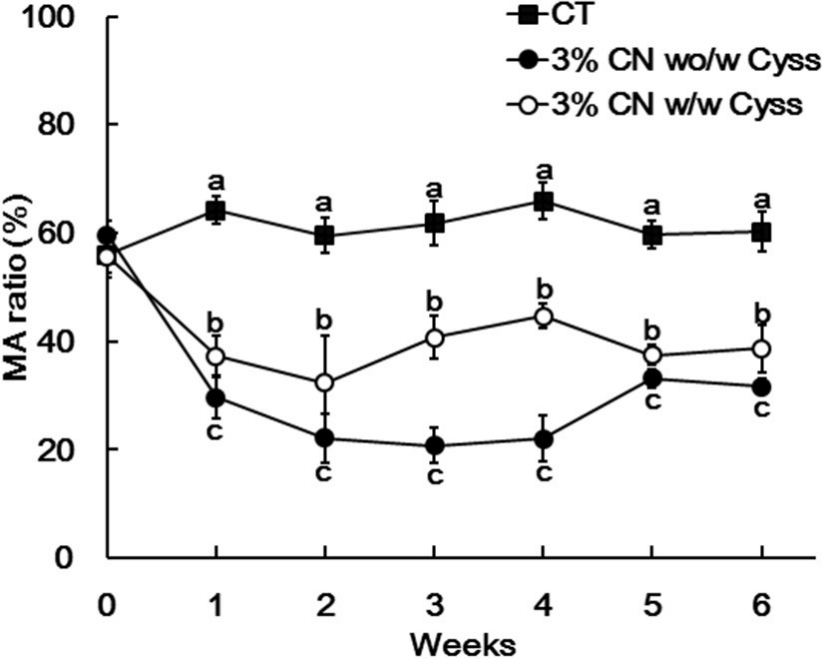
Plasma mercaptalbumin ratio in Experiment 2. Plasma samples, obtained weekly, were subjected to HPLC to determine albumin redox state. Ratios of mercaptalbumin (MA) among total albumin are shown as means ± SD (*n* = 6); values with different letters are significantly different at each time point (*P* < 0.05). CN, CT, and Cyss denote casein, control, and cystine, respectively.

Plasma albumin redox state was also determined during the experimental period. Similar to Experiment 1, the ratio of MA was significantly lower in the 3% CN wo/w Cyss diet group than the 3% CN w/w Cyss diet group during the first 4 weeks (Figure 6). When both groups were allowed to consume the 3% CN + Cyss diet *ad libitum* during the following 2 weeks, the gaps in the MA ratio between them narrowed, although the statistical significances were not dissolved until the end of the experimental period.

#### Hepatic Gene Expression

Albumin and 4E-BP1 gene expression was examined at the end of the experimental period (at 6 weeks). Compared with the CT diet group, albumin gene was suppressed (CT: 1.00 ± 0.32, 3% CN wo/w Cyss: 0.40 ± 0.06, 3%CN w/w Cyss: 0.38 ± 0.07, arbitrary unit) and 4E-BP1 gene was induced suppressed (CT: 1.00 ± 0.24, 3% CN wo/w Cyss: 2.62 ± 0.37, 3% CN w/w Cyss: 2.48 ± 0.25, arbitrary unit) in the LP diet groups. Neither of the gene expression levels differed significantly between these two LP diet groups.

### Experiment 3

To investigate how the redox state of plasma albumin in rats fed 3% CN would be modulated by dietary Cys in case it was supplementation in the form of an antioxidant, GSH/GSSG, a pilot study was conducted by feeding rats 3% CN, 3% CN + Cyss, 3% CN + GSH, 3% CN +GSSG, or 3% CN + Glu/Cyss/Gly diet, *ad libitum* for 4 weeks.

#### Dietary Intake and Body Weight

No significant difference in energy and protein intakes was seen between the LP diet groups (Supplementary Table 1), but the 3% CN diet group showed the lowest energy and protein intakes among them.

Similarly, while there was no significant difference in body weight between the LP diet groups at week 4, the 3% CN diet group exhibited the lowest body weight among them (Supplementary Table 1).

#### Glutathione Level in Liver

As GSH is predominantly synthesized and metabolized in the liver (27), effects of these dietary supplements on hepatic glutathione status were investigated at the end of the experimental period. GSH, GSSG, and GSH + GSSG levels were the lowest in the 3% CN diet group, and the differences were significant compared with the CT diet group (Table 4). All the supplementation narrowed the gap, reaching almost the same levels as the CT diet group. Similarly, the ratio of GSH/GSSG, a potential marker of cellular redox status (28), was decreased in the 3% CN diet group and was significantly lower than the CT diet group. All the supplementation increased the ratio, rising to that of the CT diet group.

**Table 4:** Hepatic glutathione status in Experiment 3.

	**CT**	**3% CN**	**3% CN + Cyss**	**3% CN + GSH**	**3% CN + GSSG**	**3% CN + Glu/Cyss/Gly**
GSH + GSSG (μmol/g liver)	5.20 ± 0.28^a^	0.67 ± 0.02^b^	3.12 ± 0.27^ab^	3.83 ± 1.51^a^	3.42 ± 0.28^a^	4.57 ± 2.24^a^
GSH (μmol/g liver)	5.04 ± 0.19^a^	0.64 ± 0.02^b^	3.02 ± 0.29^b^	3.76 ± 1.49^a^	3.37 ± 0.28^a^	4.44 ± 2.25^a^
GSSG (μmol/g liver)	0.08 ± 0.05^a^	0.01 ± 0.00^b^	0.05 ± 0.01^ab^	0.04 ± 0.01^ab^	0.03 ± 0.01^ab^	0.06 ± 0.00^ab^
GSH/GSSG	73.7 ± 31.4^ab^	48.9 ± 6.0^b^	62.5 ± 20.5^ab^	100.3 ± 23.2^ab^	124.9 ± 26.8^a^	71.9 ± 40.6^ab^

*Liver samples, obtained at the end of the end of the experimental period, were analyzed. Data are expressed as means ± SD (n = 3), and values with different superscript letters are significantly different (P < 0.05). CN, CT, Cyss, Glu, Gly GSH, and GSSG denote casein, control, cystine, glutamic acid, glycine, glutathione, and glutathione disulfide, respectively*.

#### Plasma Albumin and Redox State of Plasma Albumin

Plasma samples were obtained at the end of the experimental period (at week 4), and their albumin levels were determined (Table 5). Although no significant difference was observed between the LP diet groups, the 3% CN diet group had the lowest level among them.

**Table 5:** Plasma albumin level and mercaptalbumin ratio in Experiment 3.

	**CT**	**3% CN**	**3% CN + Cyss**	**3% CN + GSH**	**3% CN + GSSG**	**3% CN + Glu/Cyss/Gly**
Plasma albumin (g/L)	46.3 ± 4.7^a^	28.2 ± 1.3^b^	27.6 ± 2.7^b^	29.2 ± 1.4^b^	30.2 ± 1.7^b^	29.6 ± 1.0^b^
Mercaptalbumin ratio (%)	58.7 ± 3.1^a^	14.2 ± 3.6^c^	27.1 ± 1.8^b^	29.9 ± 0.8^b^	31.9 ± 1.8^b^	32.2 ± 9.8^b^

*Plasma samples, obtained at the end of the experimental period, were analyzed. Data are expressed as means ± SD (n = 3), and values with different superscript letters are significantly different (P < 0.05). CN, CT, Cyss, Glu, Gly GSH, and GSSG denote casein, control, cystine, glutamic acid, glycine, glutathione, and glutathione disulfide, respectively*.

Plasma albumin redox state was also analyzed for these samples (Table 5). MA ratio of the 3% CN diet group was the lowest and even significantly lower than the other LP diet groups.

#### Hepatic Gene Expression

Hepatic gene expression of albumin and 4E-BP1 was investigated at the end of the experimental period (Supplementary Table 2). Albumin gene was suppressed and 4E-BP1 gene was induced in the LP diet groups compared with the CT diet group. No significant difference in albumin gene expression was observed between the LP diet groups, while 4E-BP1 gene expression levels differed significantly between the LP diet groups; the level of the 3% CN diet group was significantly higher than those of the other LP diet groups except the 3% CN + Cyss diet group.

## Discussion

We demonstrated that the redox state of plasma albumin reflect albumin turnover including albumin synthesis rate, and is therefore influenced by the quantity of dietary protein (13–15). Furthermore, it was suggested our recent study that plasma albumin redox state could also be modulated by the quality of dietary protein (16). Here, aiming to elaborate how the redox state of plasma albumin is affected by protein quality in the diet such as amino acid balance, growing rats were placed on various kinds of AIN-93G-based LP diets and albumin redox state in plasma was compared.

Dietary protein sources, CN, WP, and WG, which are of either animal or plant origin with quite different amino acid compositions and protein qualities (19–24), were tested in Experiment 1. Both plasma albumin level and MA ratio was the lowest in the 3% CN diet group. Notably, the decrease in MA ratio was quite prominent, and the ratio was significantly lower than that of the 3% WP or 3% WG diet group. The 3% CN + Cyss diet group also showed lower MA ratio compared with the 3% WP or 3% WG diet group, but the ratio was still significantly higher than that of the 3% CN diet group. When considering the fact that Cys content in CN is insufficient for the growth and maintenance of rats (17), and that these LP diet-fed groups were maintained on the same amounts of energy and protein intakes controlled by a pair-feeding regimen, the decreased MA ratio in the 3% CN diet group could be primarily attributed to low Cys content in the diet. This notion may be further supported by the observation in Experiment 2, where the dietary regimen, switched from the 3% CN diet to the 3% CN + Cyss diet, caused a swift increase in the MA ratio. Thus, the severely decreased MA ratio in the 3% CN diet-fed animals would reflect a shortage of sulfur amino acids in CN, especially of Cys. Furthermore, while it has not been assessed whether supplementations of amino acids other than Cyss would modulate plasma albumin redox state, Experiment 3 showed that Glu/Cyss/Gly supplementation shifted to more reduced state than Cyss supplementation only, although the difference did not reach statistical insignificance. This result implied that Glu and/or Gly supplementation might affect plasma albumin redox state. Collectively, it can be speculated that the redox state of plasma albumin could reflect the amino acid balance of dietary protein, one of the features of protein quality. Further studies are warranted to substantiate the relationship between amino acid balance in dietary protein and plasma albumin redox state, by testing whether supplementation of respective limiting amino acids in various protein sources would modulate plasma albumin redox state, as in the case of Cyss supplementation to CN.

If it is the case that plasma albumin redox state reflects protein quality, the observation that ingesting 3% CN diet shifted plasma albumin redox state to a more oxidized state compared with that of the 3% WG diet may contradict previous reports that CN is superior to WG in protein quality as assessed by the IAAO method in rats (23, 24). The concentrations of CN and WG in the diets of these studies were 4.3–25.8 and 7.2–25.2%, respectively, while in this study the concentration was adjusted to 3%. Besides, it has been reported that ileal digestibility in pigs increased as concentration of the protein source in the diet decreased (29). It was therefore speculated that amino acid balance rather than protein digestibility might be a dominant factor into specifying protein quality in the case that protein sources are provided to growing rats in LP diet-form with a concentration as low as 3%. Furthermore, in the case where the redox state of plasma albumin in growing rats is leveraged as a bioassay for assessing protein quality, as with the IAAO method (23, 24), it would be necessary to figure out the ranges of protein source concentration that properly reflect protein quality, which requires further investigation.

Although albumin turnover, such as albumin synthesis rate, is likely involved in the shift of plasma albumin redox state in response to the amount of protein intake (14), albumin synthesis rate was not measured in this study. Instead, plasma free amino acid levels (substrates of albumin synthesis) and hepatic gene expression related to albumin synthesis was investigated to help understand its synthesis rate. Plasma free total and essential amino acid levels were lower in the LP diet groups than the CT diet groups, which was similar to the observations in our previous studies (13–15). However, when these levels were compared between the LP diet groups in Experiment 1, they did not seem to be associated with plasma albumin level or MA ratio. Thus, although plasma free amino acid levels are influenced by immediate intakes of dietary proteins and amino acids (26), they would reflect differences in the quality of dietary protein sources to limited extents in this experimental model. Expressions of two genes, albumin and 4E-BP1, were assayed in the livers; albumin synthesis is regulated primarily at the transcriptional level and its gene expression is down-regulated under protein-deficient conditions (2); dietary Cys insufficiency increased 4E-BP1 expression at both the transcriptional and translational levels, thereby suppressing global protein translation including albumin (16). Albumin gene expression was suppressed and 4E-BP1 gene expression was induced in the LP diet groups compared with the CT diet groups. In Particular, albumin gene expression levels were generally in the order of MA ratios in Experiment 1; the 3% CN diet group showed the lowest level of albumin gene expression, and the level was significantly lower than those of the other LP diet groups. In Experiment 2, the switch of dietary regimen, from the 3% CN diet to the 3% CN + Cyss diet, narrowed the gap of MA ratios and hepatic albumin gene expression between these two diet groups, observed in Experiment 1. These results can be interpreted as follows; a shortage of Cys in CN would be responsible for severe suppression of albumin gene expression in animals fed the 3% CN diet, and Cyss added to the diet partially dissolved the severity of the suppression. In the case of 4E-BP1, the gene expression was up-regulated in the LP diet groups compared with the CT diet group, but did not differ significantly between the LP diet groups in Experiment 1. Namely, under the condition where dietary protein sources are given to growing rats as a 3% protein source diet, albumin gene suppression rather than 4E-BP1 gene induction would be responsible for decreased albumin synthesis, as manifested by the shifts of plasma albumin redox state to a more oxidized state in the LP diet groups.

GSH is a Cys-containing antioxidative tripeptide (25), and bioavailability and metabolic fate of dietary GSH have not been fully elucidated. It has generally been regarded that GSH would be catabolized to γ-glutamyl amino acid, Cys, and Gly by ecto-enzymes such as γ-glutamyl transferase and dipeptidase at the brush border membrane before being absorbed in the intestine (25), while recent studies have shown the possibility that GSH could be transported across intestinal epithelial cells in intact form (30, 31). Thus, it was hypothesized that supplementing 3% CN diet with GSH would shift the plasma albumin redox state to a more reduced state compared with Cyss supplementation in a synergetic manner both by serving as a source of Cys and by exerting its antioxidative activity. In Experiment 3, which was conducted on a pilot scale, plasma albumin redox state was shifted significantly to more reduced state in rats fed the 3% CN diet with supplements, Cyss, GSH, GSSG, or Glu/Cyss/Gly, compared with rats maintained on the 3% CN diet. However, no significant difference was seen between these supplement groups. Similarly, hepatic GSH, GSSG, and GSH + GSSG levels, which were attenuated in the 3% CN diet group, were partially but significantly dissolved by all the supplements, but differences seen between these supplement groups were insignificant. Furthermore, neither hepatic albumin nor 4E-BP1 gene expression levels were different between the supplement groups. Taken together, all the supplementation to the 3% CN diet impacted plasma albumin redox state to similar extents, and GSH or GSSG added to the 3% CN diet would serve primarily as a source of Cys that is insufficient in CN, rather than exhibiting antioxidative activity. This notion may be further supported by the observation in our previous study that oxidative stress was irrelevant to plasma albumin redox state and vice versa in the LP diet model in growing rats (14). Still, it may be notable that GSSG was the only supplement that significantly alleviated the LP diet-induced decrease in the hepatic GSH/GSSG ratio, which has been measured as an index for cellular redox state (28). This trend held true for GSH supplementation, although the difference was not statistically significant. Thus, dietary GSH/GSSS intake might contribute to modulating the cellular redox state to a greater extent than Cys only or their constituting amino acids, Glu/Cyss/Gly. It should be borne in mind that it was merely tested on a pilot scale, but that efficacy of GSH/GSSG as an antioxidants have been substantiated to date by many studies (25). It should also be noted that Cys were provided as Cyss in this study, requiring reducing power to convert Cyss to Cys before being utilized for protein synthesis, which might confound the discussion in the context of antioxidantive potential.

In conclusion, the redox state of plasma albumin was shifted to a more oxidized state in rats when they were maintained on an LP diet with poor amino acid balance such as shortage of Cys in CN, and is possibly to reflect the amino acid balance of dietary protein, one of the features of protein quality, which could be useful as a biomarker that contributes to prevention of protein under-nutriton, accompanied by not only insufficient protein intake but also ingestion of poor-quality protein. However, the relationship between plasma albumin redox state and protein quality has not been fully established, as it was assessed by supplementation of single limiting amino acid (Cyss supplementation to CN), which was conducted only under a specific protein-depleted condition. Besides, this experimental condition is quite severe in nutritional sense (maintaining growing animals on diets with very low protein content), and the observation could be applied to limited cases of human nutrition. Further extensive studies are thus required to elucidate the relationship, such as by testing other protein sources with different amino acid balance and examining effects of their limiting amino acid supplementation, comparing proteins originated from an identical source with different protein digestibility, and trying other animal models such as feeding adult animals feeding diets with graded protein levels (15).

## Author Contributions

YW, X, NS, HI, TS, and YT designed the study. YW, X, YKo, MT, and YKi performed experiments and analyzed data. YW and X wrote the paper. YW and X contributed equally to this work, and YW had primary responsibility for the final content. All authors read and approved the final manuscript.

### Conflict of Interest Statement

All authors are the employees of Morinaga Milk Industry Co., Ltd.

## Abbreviations

CN: casein
CT: control
Cys: cysteine
Cyss: cystine
Glu: glutamic acid
Gly: glycine
GSH: glutathione
GSSG: glutathione disulfide
LP: low protein
MA: mercaptalbumin
NA: non-mercaptalbumin
WG: wheat gluten
WP, whey protein, 4E-BP1: an eukaryotic initiation factor 4E-binding protein 1.

## References

[1] Quinlan GJ Martin GS Evans TW. Albumin: biochemical properties and therapeutic potential. Hepatology (2005) 41:1211–9. 10.1002/hep.20720 15915465

[2] Wada Y Takeda Y Kuwahata M. Potential role of amino acid/protein nutrition and exercise in serum albumin redox state. Nutrients (2018) 10:E17. 10.3390/nu10010017 29295548 PMC5793245

[3] Kubota K Nakayama A Takehana K Kawakami A Yamada N Suzuki E. A simple stabilization method of reduced albumin in blood and plasma for the reduced/oxidized albumin ratio measurement. Int J Biomed Sci. (2009) 5:293–301. 23675150 PMC3614789

[4] Fukushima H Miwa Y Shiraki M Gomi I Toda K Kuriyama S. Oral branched-chain amino acid supplementation improves the oxidized/reduced albumin ratio in patients with liver cirrhosis. Hepatol Res. (2007) 37:765–70. 10.1111/j.1872-034X.2007.00123.x 17573945

[5] Setoyama H Tanaka M Nagumo K Naoe H Watanabe T Yoshimaru Y. Oral branched-chain amino acid granules improve structure and function of human serum albumin in cirrhotic patients. J Gastroenterol. (2017) 52:754–65. 10.1007/s00535-016-1281-2 27873095 PMC5437197

[6] Terawaki H Yoshimura K Hasegawa T Matsuyama Y Negawa T Yamada K. Oxidative stress is enhanced in correlation with renal dysfunction: examination with the redox state of albumin. Kidney Int. (2004) 66:1988–93. 10.1111/j.1523-1755.2004.00969.x 15496170

[7] Regazzoni L Del Vecchio L Altomare A Yeum KJ Cusi D Locatelli F. Human serum albumin cysteinylation is increased in end stage renal disease patients and reduced by hemodialysis: mass spectrometry studies. Free Radic Res. (2013) 47:172–80. 10.3109/10715762.2012.756139 23215783

[8] Era S Kuwata K Imai H Nakamura K Hayashi T Sogami M. Age-related change in redox state of human serum albumin. Biochim Biophys Acta (1995) 1247:12–6. 10.1016/0167-4838(94)00166-E 7873580

[9] Oettl K Reibnegger G Schmut O. The redox state of human serum albumin in eye diseases with and without complications. Acta Ophthalmol. (2011) 89:e174–9. 10.1111/j.1755-3768.2009.01824.x 20064117

[10] Imai H Era S Hayashi T Negawa T Matsuyama Y Okihara K Effect of propolis supplementation on the redox state of human serum albumin during high-intensity kendo training. Adv Exerc Sports Physiol. (2005) 11:109–13.

[11] Lamprecht M Greilberger JF Schwaberger G Hofmann P Oettl K. Single bouts of exercise affect albumin redox state and carbonyl groups on plasma protein of trained men in a workload-dependent manner. J Appl Physiol. (2008) 104:1611–7. 10.1152/japplphysiol.01325.2007 18420715

[12] Lamprecht M Oettl K Schwaberger G Hofmann P Greilberger JF. Protein modification responds to exercise intensity and antioxidant supplementation. Med Sci Sports Exerc. (2009) 41:155–63. 10.1249/MSS.0b013e31818338b7 19092694

[13] Kuwahata M Hasegawa M Kobayashi Y Wada Y Kido Y. An oxidized/reduced state of plasma albumin reflects malnutrition due to an insufficient diet in rats. J Clin Biochem Nutr. (2017) 60:70–5. 10.3164/jcbn.16-33 28163385 PMC5281528

[14] Wada Y Sato Y Miyazaki K Takeda Y Kuwahata M. The reduced/oxidized state of plasma albumin is modulated by dietary protein intake partly via albumin synthesis rate in rats. Nutr Res. (2017) 37:46–57. 10.1016/j.nutres.2016.12.003 28215314

[15] Wada Y Komatsu Y Izumi H Shimizu T Takeda Y Kuwahata M. Increased ratio of non-mercaptalbumin−1 among total plasma albumin demonstrates potential protein undernutrition in adult rats. Front Nutr. (2018) 5:64. 10.3389/fnut.2018.00064 30090810 PMC6068262

[16] Kuwahata M Kobayashi Y Wada Y Aoi W Kido Y. Dietary cystine is important to maintain plasma mercaptalbumin levels in rats fed low-protein diets. Nutr Res. (2018) 56:79–89. 10.1016/j.nutres.2018.04.019 30055777

[17] Reeves PG. Components of the AIN−93 diets as improvements in the AIN−76A diet. J Nutr. (1997) 127(5 Suppl.):838s−41s. 10.1093/jn/127.5.838S 9164249

[18] Boirie Y Dangin M Gachon P Vasson MP Maubois JL Beaufrère B. Slow and fast dietary proteins differently modulate postprandial protein accretion. Proc Natl Acad Sci USA. (1997) 94:14930–5. 10.1073/pnas.94.26.14930 9405716 PMC25140

[19] Hall WL Millward DJ Long SJ Morgan LM. Casein and whey exert different effects on plasma amino acid profiles, gastrointestinal hormone secretion and appetite. Br J Nutr. (2003) 89:239–48. 10.1079/BJN2002760 12575908

[20] Deglaire A Bos C Tomé D Moughan PJ. Ileal digestibility of dietary protein in the growing pig and adult human. Br J Nutr. (2009) 102:1752–9. 10.1017/S0007114509991267 19706206

[21] Cervantes-Pahm SK Stein HH. Ileal digestibility of amino acids in conventional, fermented, and enzyme-treated soybean meal and in soy protein isolate, fish meal, and casein fed to weanling pigs. J Anim Sci. (2010) 88:2674–83. 10.2527/jas.2009-2677 20407072

[22] Rutherfurd SM Fanning AC Miller BJ Moughan PJ. Protein digestibility-corrected amino acid scores and digestible indispensable amino acid scores differentially describe protein quality in growing male rats. J Nutr. (2015) 145:372–9. 10.3945/jn.114.195438 25644361

[23] Ogawa A Naruse Y Shigemura Y Kobayashi Y Suzuki I Wada S. An evaluation of protein intake for metabolic demands and the quality of dietary protein in rats using an indicator amino acid oxidation method. J Nutr Sci Vitaminol. (2011) 57:418–25. 10.3177/jnsv.57.418 22472284

[24] Ogawa A Murayama H Hayamizu K Kobayashi Y Kuwahata M Kido Y. A simple evaluation method for the quality of dietary protein in rats using an indicator amino acid oxidation technique. J Nutr Sci Vitaminol. (2015) 61:123–30. 10.3177/jnsv.61.123 26052142

[25] Lu SC. Glutathione synthesis. Biochim Biophys Acta (2013) 1830:3143–53. 10.1016/j.bbagen.2012.09.008 22995213 PMC3549305

[26] Young VR Marchini JS Cortiella J. Assessment of protein nutritional status. J Nutr. (1990) 120(Suppl. 11):1496–502. 10.1093/jn/120.suppl_11.1496 2243295

[27] Ookhtens M Kaplowitz N. Role of the liver in interorgan homeostasis of glutathione and cyst(e)ine. Semin Liver Dis. (1998) 18:313–29. 10.1055/s-2007-1007167 9875551

[28] Wu G Fang YZ Yang S Lupton JR Turner ND. Glutathione metabolism and its implications for health. J Nutr. (2004) 134:489–92. 10.1093/jn/134.3.489 14988435

[29] Zhai H Adeola O. Apparent and standardized ileal digestibilities of amino acids for pigs fed corn- and soybean meal-based diets at varying crude protein levels. J Anim Sci. (2011) 89:3626–33. 10.2527/jas.2010-3732 21724945

[30] Park EY Shimura N Konishi T Sauchi Y Wada S Aoi W. Increase in the protein-bound form of glutathione in human blood after the oral administration of glutathione. J Agric Food Chem. (2014) 62:6183–9. 10.1021/jf501338z 24877771

[31] Kovacs-Nolan J Rupa P Matsui T Tanaka M Konishi T Sauchi Y. *In vitro* and *ex vivo* uptake of glutathione (GSH) across the intestinal epithelium and fate of oral GSH after *in vivo* supplementation. J Agric Food Chem. (2014) 62:9499–506. 10.1021/jf503257w 25198144

