# Supplementary material for: Plasma Albumin Redox State Is Responsive to the Amino Acid Balance of Dietary Proteins in Rats Fed a Low Protein Diet
Source: Front Nutr. 2019 Feb 15;6:12. doi: 10.3389/fnut.2019.00012 (PMC6385526; doi:10.3389/fnut.2019.00012)
Supplement: Supplementary file 1 [file Data_Sheet_1.docx]

**Supplementary Table 1. Energy/protein intakes and body weights in Experiment 3.**

|  | **CT** | **3% CN** | **3% CN +**  **Cyss** | **3% CN +**  **GSH** | **3% CN + GSSG** | **3% CN +**  **Glu/Cyss/Gly** |
| --- | --- | --- | --- | --- | --- | --- |
| Energy Intake  (kJ/d) | 210.0 ± 19.2^a^ | 110.0 ± 4.8^b^ | 118.4 ± 10.0^b^ | 123.6 ± 14.0^b^ | 125.9 ± 1.5^b^ | 115.4 ± 4.8^b^ |
| Protein Intake  (g/d) | 2.47 ± 0.23^a^ | 0.20 ± 0.01^b^ | 0.21 ± 0.02^b^ | 0.22 ± 0.03^b^ | 0.23 ± 0.00^b^ | 0.22 ± 0.01^b^ |
| Body Weight  (g) |  |  |  |  |  |  |
| Week 0 | 80.1 ± 8.4 | 81.0 ± 4.0 | 80.5 ± 6.9 | 80.5 ± 5.7 | 80.8 ± 3.2 | 80.6 ± 6.2 |
| Week 4 | 218.1 ± 18.3^a^ | 70.1 ± 2.4^b^ | 83.9 ± 6.7^b^ | 82.5 ± 7.2^b^ | 83.6 ± 2.6^b^ | 82.2 ± 1.3^b^ |

Data are expressed as means ± SD (*n* = 3), and values with different superscript letters are significantly different (*P* < 0.05). CN, CT, Cyss, Glu, Gly GSH, and GSSG denote casein, control, cystine, glutamine, glycine, glutathione, and glutathione disulfide, respectively.

**Supplementary Table 2. Hepatic albumin and 4E-BP1 gene expression in Experiment 3.**

|  | CT | 3% CN | 3% CN +  Cyss | 3% CN +  GSH | 3% CN +  GSSG | 3% CN +  Glu/Cyss/Gly |
| --- | --- | --- | --- | --- | --- | --- |
| Gene (AU) |  |  |  |  |  |  |
| Albumin | 1.00 ± 0.08^a^ | 0.42 ± 0.15^b^ | 0.24 ± 0.06^b^ | 0.35 ± 0.05^b^ | 0.34 ± 0.10^b^ | 0.29 ± 0.09^b^ |
| 4E-BP1 | 1.00 ± 0.04^c^ | 3.88 ± 0.53^a^ | 2.95 ± 0.22^ab^ | 2.82 ± 0.51^b^ | 2.62 ± 0.17^b^ | 2.70 ± 0.37^b^ |

Data are expressed as means ± SD (*n* = 3), and values with different superscript letters are significantly different (*P* < 0.05). CN, CT, Cyss, Glu, Gly GSH, and GSSG denote casein, control, cystine, glutamine, glycine, glutathione, and glutathione disulfide, respectively.
